# Lipoprotein(a) Is Associated With Increased Risk of Abdominal Aortic Aneurysm

**DOI:** 10.1016/j.jacbts.2025.101457

**Published:** 2026-01-06

**Authors:** Pranav Sharma, Renae Judy, Shuai Yuan, Corry Gellatly, Katie L. Saxby, Daniel J. Rader, Matthew J. Bown, Michael G. Levin, Scott M. Damrauer

**Affiliations:** a Department of Surgery, Perelman School of Medicine, University of Pennsylvania, Philadelphia, Pennsylvania, USA; b Department of Medicine, Washington University School of Medicine, Washington University in St. Louis, Saint Louis, Missouri, USA; c Department of Cardiovascular Sciences and NIHR Leicester Biomedical Research Centre, University of Leicester, Glenfield General Hospital, Leicester, United Kingdom; d Department of Medicine, Perelman School of Medicine, University of Pennsylvania, Philadelphia, Pennsylvania, USA; e Department of Genetics, University of Pennsylvania, Philadelphia, Pennsylvania, USA; f Corporal Michael J. Crescenz VA Medical Center, Philadelphia, Pennsylvania, USA; g Division of Cardiovascular Medicine, Department of Medicine, University of Pennsylvania Perelman School of Medicine, Philadelphia, Pennsylvania, USA

**Keywords:** abdominal aortic aneurysm, apolipoprotein B, genetic epidemiology, lipoprotein(a), mendelian randomization

## Abstract

Lp(a) is a genetically determined lipoprotein targeted by emerging therapies. In a UK Biobank analysis (1,026 abdominal aortic aneurysm [AAA] cases, 469,989 controls), elevated Lp(a) was associated with increased risk of AAA, including at clinically relevant thresholds while controlling for traditional risk factors, including ApoB. Multivariable Mendelian randomization confirmed a causal relationship between lipoprotein(a) [Lp(a)] and AAA independent of apolipoprotein B. These findings support Lp(a) as a modifiable risk factor and potential therapeutic target for AAA, a condition with limited medical treatment options. AAA should be considered as an outcome in future clinical trials of Lp(a)-lowering therapies.

## Highlights

• Elevated Lp(a) levels were associated with increased risk of AAA, independent of traditional cardiovascular risk factors, including ApoB.
• Using both observational and genetic analyses, we demonstrate an ApoB-independent association between Lp(a) and AAA risk.
• Elevated Lp(a) levels above guideline (≥125 nmol/L) and trial-based (≥150 nmol/L) thresholds were associated with nearly 2-fold higher risk of AAA (OR: 1.82 and 1.93, respectively; both *P <* 0.001), compared with individuals below these thresholds.
• These findings suggest that Lp(a) lowering may offer therapeutic benefit for AAA management, independent of conventional risk factor modification, including ApoB reduction.

## Visual Abstract

**Figure:**
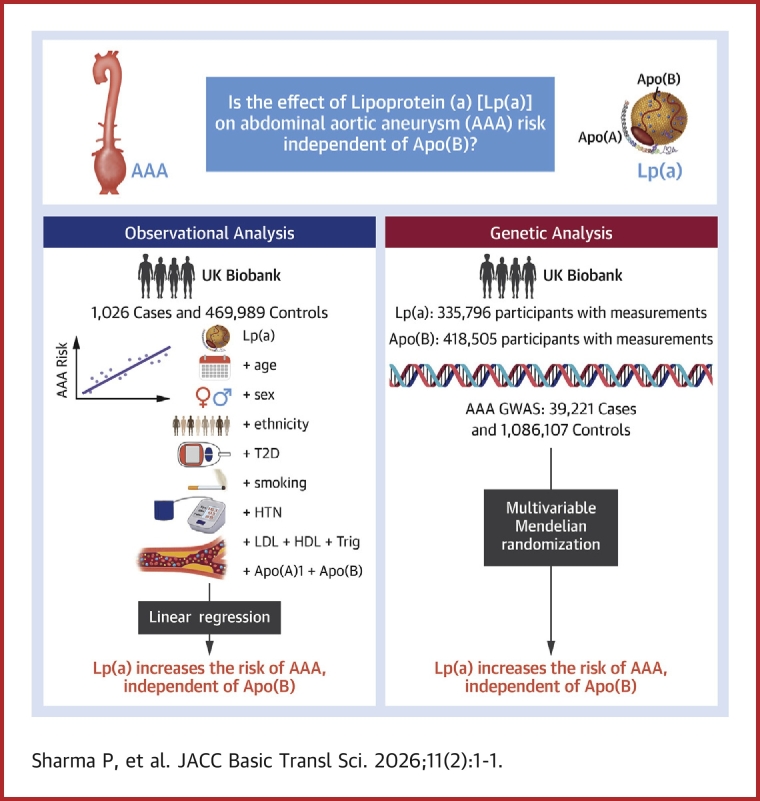

Abdominal aortic aneurysm (AAA) lacks effective medical therapies to prevent progression or rupture. Current management focuses on longitudinal monitoring until aneurysm size reaches the point at which the risk of rupture exceeds the risk of repair.^1^ Multiple pharmacological therapies, including angiotensin-converting enzyme inhibitors, angiotensin receptor blockers, and matrix metalloproteinase inhibitors, showed promise in AAA preclinical models but have not been effective in reducing aneurysm growth or rupture and are not recommended for treating AAA.^2^, ^3^, ^4^, ^5^, ^6^

The high failure rate of candidate therapeutics has prompted interest in genetically validated targets, which are more likely to succeed in clinical development.^7^^,^^8^ Recent large-scale efforts have identified inflammation and atherogenesis as key processes underlying the development of AAA, and identified genetic variants at the lipoprotein(a) locus that confer increased susceptibility to AAA.^9^^,^^10^ Lipoprotein(a) [Lp(a)] is an apolipoprotein B (ApoB)–containing molecule that has been observationally associated with atherosclerotic cardiovascular disease through putative mechanisms including inflammation, thrombosis, calcification, and atherogenesis.^11^ Lp(a) is one of the most heritable human traits, and is strongly associated with multiple atherosclerotic cardiovascular diseases (ASCVDs).^11^ With the emergence of therapies targeting Lp(a),^12^ we sought to further explore this relationship in the context of AAA pathogenesis.

We sought to investigate the role of Lp(a) in AAA pathogenesis through both observational and genetic analyses. Leveraging large-scale observational data from UK Biobank (UKB), we examined the relationship between Lp(a) levels and AAA risk, while accounting for established risk factors. Additionally, we utilized genetic evidence, employing multivariable Mendelian randomization (MVMR) to evaluate the association of Lp(a) with AAA in conjunction with ApoB. Mendelian randomization (MR) leverages the natural randomization of risk-factor associated alleles to mimic the results of randomized clinical trials to minimize bias and reverse causality that may confound typical observational study designs, contingent upon key assumptions.^13^^,^^14^ Using MVMR allows for the simultaneous assessment of multiple exposures, providing a more comprehensive understanding of their independent effects on AAA risk. This comprehensive approach not only enhances our understanding of AAA pathophysiology but also highlights the potential of Lp(a) as a therapeutic target, offering new avenues for noninvasive intervention.

## Methods

The study comprised 2 components. The first was an observational analysis to test the association of measured Lp(a) with AAA while controlling for ApoB and traditional AAA risk factors using data from the UKB. The second was a causal inference experiment, using MVMR to test for a putatively causal association between Lp(a) and AAA while controlling for ApoB. Our analysis was performed in accordance with the STROBE-MR guidelines.^15^ All participants in the underlying studies gave written consent to the use of their data and those studies were approved by the relevant Institutional Review Board.

### Observational analysis of Lp(a) and AAA

#### Study design and setting

We conducted a cross-sectional cohort analysis using data from the UKB, which comprises over 500,000 individuals aged 40 to 69 years, enrolled between 2004 and 2010.^16^ Our analysis was restricted to the subset of individuals with measured levels of Lp(a) and ApoB. All Lp(a) and ApoB measurements were performed using the same analytic platform and framework. UKB applied extensive quality control and drift correction algorithms, to minimize batch-related variability and ensure assay consistency across all participants. Further details regarding QC of biomarker samples are included online.^17^

Serum Lp(a) concentration, as a continuous, raw measurement was the primary exposure. Serum Lp(a) concentrations in UKB were measured using an immuno-turbidimetric assay (Assay Manufacturer: Randox Bioscience, UK; Analytic Platform: Beckman Coulter AU5800) with an analytic range of 5.76 to 189 nmol/L.^18^ For Lp(a) values outside of the normal range, there were a total of 82,543 values recorded. Of these values, 48,354 (58.6%) were below 3.8 nmol/L and 34,189 (41.4%) were above 189 nmol/L. Values falling outside the assay's reportable range, those >189 or <3.8 nmol/L were set to the respective boundary values (189 and 3.8 nmol/L) for analysis. We also tested the association of clinically elevated levels of Lp(a), using thresholds above 125 nmol/L, based on current American College of Cardiology (ACC)/American Heart Association (AHA)/European Atherosclerosis Society (EAS) guidelines^19^^,^^20^ and above 150 nmol/L, consistent with the inclusion criteria of the phase 2 and 3 OCEAN(a) DOSE and Lp(a)HORIZON clinical trial,^21^^,^^22^ with AAA.

Covariates included ApoB, age, sex, race/ethnicity, diabetes status, smoking status, hypertension, ApoA1 concentration, low-density lipoprotein (LDL) cholesterol, high-density lipoprotein (HDL) cholesterol, and triglycerides. Data fields from the UKB data dictionary can be found in Supplemental Table 1.

#### Primary outcome

The primary outcome was prevalent abdominal aortic aneurysm as indicated by at least 1 instance of an applicable ICD-9 (International Classification of Diseases-9th Revision), ICD-10, or OPCS-4 (OPCS Classification of Interventions and Procedures) at the time of analysis. This definition captured both prevalent diagnoses at baseline and incident cases identified during follow-up. ICD-9 codes included 441.3 or 441.4. ICD-10 codes included I71.3 or I71.4. OPCS-4 codes included L184, L185, L186, L194, L195, L196, L271, L275, L276, L281, L285, L286. Data fields from the UKB data dictionary can be found in Supplemental Table 1.

#### Statistical methods

Data are presented for continuous variables using median with 25th-75th percentiles (Q1-Q3) or for categorical variables as count (percentage). Differences between groups were assessed using the Wilcoxon rank-sum test for continuous variables and the chi-square test for categorical variables. We used logistic regression to test the relationship between Lp(a) concentration and prevalent AAA. Initially, we examined a model that assessed AAA risk using Lp(a) adjusted for age, sex, and race/ethnicity. Subsequently, we extended our model to include additional covariates: ApoB concentration, ApoA1 concentration, diabetes, smoking, hypertension, LDL cholesterol, HDL cholesterol, and triglycerides. We report the OR with 95% CI of prevalent AAA for a 10-nmol/L increase in Lp(a).

We then examined the relationship between clinically elevated levels of Lp(a), defined as ≥125 nmol/L based on current ACC/AHA/EAS guidelines^10^^,^^20^ and ≥150 nmol/L, as defined by the inclusion criteria of OCEAN(a)-DOSE and Lp(a)HORIZON clinical trials.^21^^,^^22^ OCEAN(a)- DOSE criteria included participants with Lp(a) ≥150 nmol/L, while Lp(a)HORIZON selected participants with Lp(a) ≥70 mg/dL, which corresponds to approximately 150 nmol/L using a conversion factor of 2.15.^23^ ORs comparing those with elevated vs normal Lp(a) levels (<125 and <150 nmol/L, respectively) are reported with adjustment for the same covariates as highlighted in the previous text. As a sensitivity analysis, we excluded individuals with Lp(a) values outside the reportable range (>189 or <3.8 nmol/L) and repeated the previously mentioned analyses.

Finally, to further investigate the risk associated with varying Lp(a) levels, we categorized Lp(a) into 10 nmol/L bins, using the first decile of Lp(a) concentration as the reference group. This approach, while controlling for covariates, allowed us to visualize the risk of AAA across the distribution of Lp(a) and assess for potential threshold effects at higher concentrations. In parallel, we applied restricted cubic spline models using the “splines” package in R to flexibly model the continuous association between Lp(a) and AAA. Knots were placed at quantiles of the Lp(a) distribution, allowing for a nonlinear fit. The spline models were adjusted for the same covariates as in the primary analysis, and results were plotted to visualize the smoothed ORs and 95% CIs across the range of Lp(a) concentrations.

All statistical analyses were computed using version 4.3.2 of R statistical software.^24^ Statistical significance was determined based on a *P* value <0.05.

### Genetic causal inference

#### Study design and setting

Multivariable MR was used to test the association between genetically determined Lp(a) levels and genetic liability to AAA, while controlling for genetically determined ApoB levels. Publicly available summary statistics were obtained from the largest genome-wide association studies (GWAS) of these traits.^9^^,^^25^

#### MR assumptions

A genetic variant can be considered as an instrumental variable for a given exposure if it satisfies the instrumental variable assumptions: 1) it is associated with the exposure; 2) it is not associated with the outcome caused by alternative confounding pathways; and 3) it does not affect the outcome except potentially through the exposure.^26^^,^^27^ By using large GWAS and evaluating r^2^ and F-statistic of the instruments, we can ensure that the first assumption is met. F-statistics >10 suggest that potential of weak instruments leading to biased associations is low, while higher r^2^ values indicate the model is explaining a larger proportion of the variance in the dependent variable.^28^ Details of our instrument selection approach are presented in the following text. The second and third assumptions cannot be affirmatively proven; however, by performing a range of pleiotropy-robust sensitivity analyses (as discussed in the following text), these assumptions may be falsified.

#### Genetic data

The primary exposure was Lp(a); we also tested the association of ApoB independently and in a joint model. Genetic instruments for Lp(a) and ApoB were developed from previously published GWAS data from the Pan-UKB project, comprising 335,796 and 418,505 UKB participants, respectively.^25^ Summary statistics are publicly available online.^29^

The primary outcome was prevalent AAA.^9^ Summary statistics for AAA were obtained from the recently published multipopulation GWAS meta-analysis by Roychowdhury et al^9^ comprising 39,221 individuals with and 1,086,107 without AAA from 14 cohorts. GWAS summary statistics for AAA are publicly available online.^30^

#### Exposure instrument selection

Independent (r^2^ < 0.001, >10,000 kb) genetic variants associated with either Lp(a) or ApoB at genome-wide significance (*P <* 5 × 10^−8^) were used to instrument the exposures. F-statistics and cumulative r^2^ were calculated for each instrument to evaluate the strength of association between the instrument and exposure.

#### Statistical methods

We first performed a univariable analysis to test the association between Lp(a) and AAA using inverse-variance weighting with multiplicative random effects. Weighted median, weighted mode, and MR Egger methods were applied as sensitivity analyses; these methods make different assumptions about the presence of pleiotropy, which may invalidate the second and third MR assumptions.^13^^,^^14^ Because Lp(a) particles contain ApoB, it is possible that the effects of Lp(a) on AAA could be mediated by ApoB. To account for this possibility, we performed MVMR, which accounts for pleiotropic effects of Lp(a)-associated variants on ApoB and estimates the direct effect of Lp(a) on AAA. Two-sample Mendelian randomization was performed using the TwoSampleMR package in R.^31^, ^32^, ^33^, ^34^ Statistical significance was determined based on a *P* value <0.05/2 = 0.025; the Bonferroni correction was used to control for multiple testing in the univariable 2 sample MR experiments.

## Results

### Observational analysis of Lp(a) and AAA

There were 471,015 UKB participants with measured Lp(a) available for analysis. Participants had a median age of 58 years at enrollment (Q1-Q3: 50-63 years), 216,553 (46.0%) were men, and 247,879 (52.6%) were ever smokers (Table 1). The study population included 1,026 individuals with AAA and 469,989 individuals without AAA. The median Lp(a) value was 19.66 nmol/L (Q1-Q3: 7.60-74.88 nmol/L). Using logistic regression, we first tested for an association between Lp(a) and AAA, controlling for age, sex, and race/ethnicity, finding a 5% increase in risk per 10-nmol/L increase in Lp(a) (OR: 1.05; 95% CI: 1.04-1.06; *P <* 0.01). This association remained robust after controlling for additional clinical risk factors and covariates, including diabetes, smoking, hypertension, ApoA1 concentration, LDL cholesterol, HDL cholesterol, and triglycerides, as well as ApoB (OR: 1.05 per 10-nmol increase of Lp(a); 95% CI: 1.04-1.06; *P <* 0.01). This analysis identified several covariates that exhibited significant associations with AAA risk (*P <* 0.01), including male sex (OR: 5.36; 95% CI: 4.27-6.73), hypertension (OR: 1.71; 95% CI: 1.48-1.96), smoking (OR: 3.21; 95% CI: 2.57-3.99), age (OR: 1.18; 95% CI: 1.16-1.20), diabetes (OR: 1.25; 95% CI: 1.06-1.46), LDL levels (OR: 0.99; 95% CI: 0.98-0.99), and ApoA1 levels (OR: 0.38; 95% CI: 0.18-0.82). Results of all covariates and their association with AAA risk are shown in Supplemental Table 2. Results of all covariates from sensitivity analyses excluding high and low Lp(a) values are shown in Supplemental Table 3.

**Table 1:** Summary From the UK Biobank Population Analytic Cohort

	Overall (N = 517,803)	Overall With Lp(a) Measured (n = 417,015)	Lp(a) <150 nmol/L (n = 412,100)	Lp(a) ≥150 nmol/L (n = 58,915)
Age at recruitment, y	58 (50 - 63)	58 (50-63)	58 (50-63)	59 (52-64)
Male	236,917 (45.8)	216,553 (46.0)	192,533 (46.7)	24,020 (40.8)
AAA Cases	1,137 (0.2)	1,026 (0.2)	803 (0.2)	223 (0.4)
AAA Controls	516,666 (99.8)	469,989 (99.8)	411,297 (99.8)	58,692 (99.6)
Diabetes	79,293 (15.3)	72,187 (15.3)	60,661 (14.7)	11,526 (19.6)
Participant who have ever smoked	271,618 (52.5)	247,879 (52.6)	216,915 (52.6)	30,964 (52.6)
Hypertension	144,134 (27.8)	131,250 (27.9)	112,857 (27.4)	18,393 (31.2)
High LDL (LDL ≥160 mg/dL)	117,932 (22.8)	114,185 (24.2)	94,589 (22.9)	19,596 (33.2)
Low HDL (HDL <40 mg/dL men, <50 mg/dL women)	95,900 (18.5)	93,179 (19.8)	83,089 (20.2)	10,090 (17.1)
High triglycerides (triglycerides ≥150 mg/dL)	195,373 (37.8)	189,768 (40.3)	167,598 (40.7)	22,170 (37.6)
Lp(a), nmol/L	19.66 (7.60-74.88)	19.66 (7.60-74.88)	15.20 (6.67-43.30)	189.00 (173.20-189.00)
LDL, mg/dL	135.5 (112.4-159.3)	136.1 (113.7-159.3)	135.2 (112.9-158.1)	144.3 (120.2-168.9)
HDL, mg/dL	54.1 (46.0-65.1)	54.1 (45.3-64.9)	53.8 (45.3-64.5)	56.1 (47.2-66.5)
Triglycerides, mg/dL	153.0 (88.6-185.3)	131.1 (92.8-190.2)	131.8 (92.9-191.4)	127.6 (91.2-181.6)
HbA1c, %	5.5 (5.0-5.7)	5.36 (5.14-5.56)	5.36 (5.13-5.55)	5.40 (5.18-5.58)
Creatinine, umol/L	71.0 (61.0-82.0)	70.5 (61.4-81.0)	70.5 (61.5-81.0)	69.9 (61.0-80.5)
ApoB, g/L	1.00 (0.90-1.20)	1.02 (0.86-1.18)	1.01 (0.86-1.17)	1.07 (0.92-1.24)
ApoA1, g/L	1.50 (1.30-1.70)	1.51 (1.35-1.70)	1.51 (1.34-1.69)	1.51 (1.38-1.73)

Values are median (Q1-Q3) or n (%). *P* values are derived from comparisons between individuals with Lp(a) ≤150 and >150 nmol/L, using the Wilcoxon rank-sum test for continuous variables and the chi-square test for categorical variables. All *P* values were <0.001 except for smoking status *(P =* 0.041).

We next evaluated threshold-based risk. Using the guideline-defined (AHA/ACC/EAS) cutoff of ≥125 nmol/L, individuals with elevated Lp(a) had significantly higher odds of AAA compared with those below the threshold (OR: 1.82; 95% CI: 1.57-2.12; *P <* 0.01). Similarly, using the ≥150 nmol/L threshold employed in clinical trials of Lp(a)-lowering therapies (eg, OCEAN(a)-DOSE and Lp(a)HORIZON), individuals above this level had 93% increased odds of AAA (OR: 1.93; 95% CI: 1.65-2.28; *P <* 0.01).

**Figure 1:**
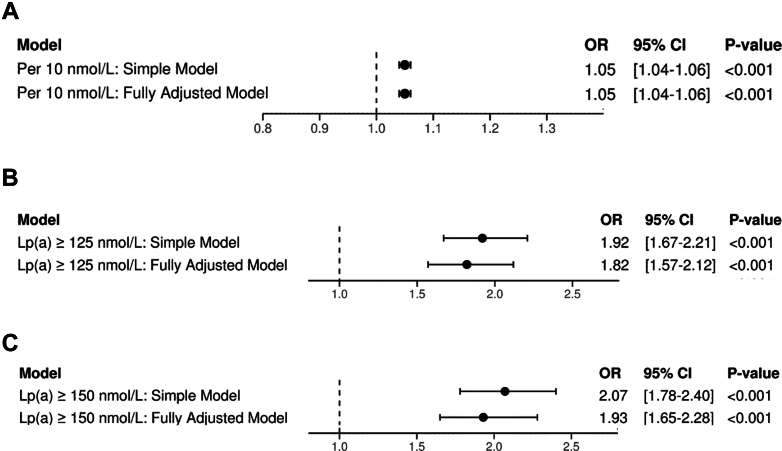
Observational Models Evaluating Lp(a) and AAA Risk (A) Per 10-nmol/L increase in lipoprotein(a) [Lp(a)] concentration was associated with progressively increased odds of abdominal aortic aneurysm (AAA), modeled using logistic regression. (B) Elevated Lp(a) above 125 nmol/L and (C) above 150 nmol/L were each associated with higher odds of AAA compared with those below these thresholds. ORs and 95% CIs are shown for both a simple model (adjusted for age, sex, and ethnicity) and a fully adjusted model (additionally adjusted for apolipoprotein [Apo] B, ApoA1, diabetes, smoking, hypertension, low-density lipoprotein cholesterol [LDL-C], high-density lipoprotein cholesterol [HDL-C], and triglycerides). These findings support a threshold-based risk model and highlight the independent association of Lp(a) with AAA.

Figure 1 summarizes these associations, including models adjusted for age, sex, and race/ethnicity, as well as fully adjusted models for each of the following: per 10 nmol/L increase in Lp(a), thresholds ≥125 nmol/L, and ≥150 nmol/L. Results from a sensitivity analysis excluding participants with high and low Lp(a) values yielded smaller effects but significant findings: per 10 nmol/L increase in Lp(a) (OR: 1.03; 95% CI: 1.02-1.05; *P <* 0.01), thresholds ≥125 nmol/L (OR: 1.36; 95% CI: 1.10-1.68; *P <* 0.01), and ≥150 nmol/L (OR: 1.38; 95% CI: 1.06-1.80; *P <* 0.01), as presented in Supplemental Figure 1.

**Figure 2:**
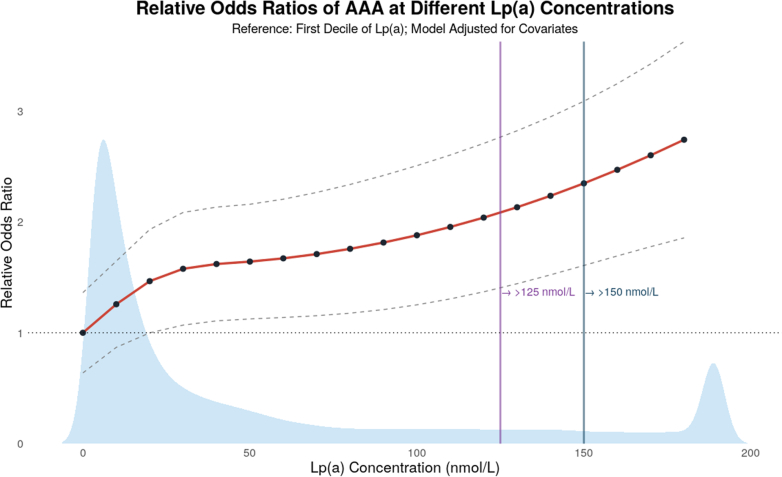
Lp(a) Dose-Response Relationship With AAA Generalized linear regression model evaluating the association between Lp(a) concentration and odds of AAA, using the first decile of Lp(a) as the reference. The red curve displays the modeled OR for AAA scaled per 10 nmol/L increase in Lp(a), with gray dashed lines representing 95% CIs. The blue histogram indicates the population distribution of Lp(a). The horizontal dotted line marks an OR of 1 at the reference level. Vertical purple and blue lines denote Lp(a) thresholds of ≥125 and ≥150 nmol/L, respectively, where AAA risk increases by 1.82- and 1.93-fold. Abbreviations as in Figure 1.

Finally, to visualize the relationship between Lp(a) and AAA, we fit a multivariable logistic regression model using natural cubic splines with 3 degrees of freedom to capture potential nonlinear effects and explore threshold behavior. Lp(a) concentrations were modeled continuously and plotted in 10 nmol/L increments, using the first decile of Lp(a) as the reference. The resulting dose-response curve suggested a largely linear association between Lp(a) and AAA risk, shown in Figure 2.

### Genetic causal inference with MR

**Figure 3:**
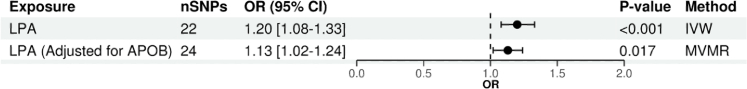
Mendelian Randomization Analyses of Lp(a) and Risk of AAA This figure presents univariable and multivariable Mendelian randomization (MVMR) analyses assessing the association between Lp(a) and AAA risk. Univariable MR was performed using the inverse variance weighted (IVW) method, while MVMR adjusted for ApoB to isolate the Lp(a)-specific effect. Results are shown as ORs with 95% CIs per 1-SD increase in biomarker level. Findings support a potential causal role of Lp(a) in AAA risk, independent of ApoB. Abbreviations as in Figure 1.

The 2-sample MR was used to test the associations of genetically determined Lp(a) and ApoB levels with genetic liability to AAA. Our genetic instruments for Lp(a) (F [mean]: 948; r^2^: 0.06) and ApoB (F [mean]: 167; r^2^: 0.08) were strongly associated with each respective exposure. After accounting for multiple testing (*P <* 0.05/2 = *P <* 0.025), both genetically determined Lp(a) and ApoB each showed significant positive associations with genetic liability to AAA in univariable analyses. The ORs were 1.20 (95% CI: 1.08-1.33; *P* < 0.001) per SD increase in genetically determined levels of Lp(a) (Figure 3). Harmonized data are shown in Supplemental Table 4. Sensitivity analyses using MR-Egger, weighted median, and weighted mode estimators demonstrated consistent directionality with the inverse variance weighted results without evidence of horizontal pleiotropy (Supplemental Table 5).

Given that Lp(a) is an ApoB-containing particle, we used MVMR to account for the potential mediating effects of ApoB and estimate the direct effect of genetically determined Lp(a) on genetic liability to AAA, independent of genetically determined ApoB. Conditional F-statistics were calculated to assess instrument strength after accounting for the correlation between Lp(a) and ApoB instruments, yielding values of 92 for Lp(a) and 146 for ApoB, indicating strong instruments and minimizing the risk of weak instrument bias. MVMR confirmed a significant association between increased genetically determined Lp(a) and an increased genetic liability to AAA independent of genetically determined ApoB (OR: 1.13 per SD increase in Lp(a); 95% CI: 1.02-1.24; *P <* 0.02) (Figure 3). Harmonized data are shown in Supplemental Table 6.

## Discussion

We conducted both observational and genetic analyses to investigate the hypothesis that circulating Lp(a) is a risk factor for AAA. We found observational evidence that elevated Lp(a) concentration was associated with a significantly increased risk of AAA, and this effect was independent of traditional cardiovascular risk factors, including ApoB. Genetic analyses further support this association, suggesting a significant association between genetically determined elevated Lp(a) and increased genetic liability to AAA, implying a causal association. These findings have important implications for Lp(a) as both a risk factor and a potential novel therapeutic target for AAA.

To contextualize the observed relationship between Lp(a) and AAA, we compared our findings to prior risk estimates from coronary and broader atherosclerotic disease outcomes. Our observational analysis demonstrates a modest increase in risk of AAA per 10-nmol/L increase in Lp(a) (OR: 1.05; 95% CI: 1.04-1.06). However, individuals with clinically elevated Lp(a) concentrations (≥125 and ≥150 nmol/L) have a substantially higher risk (OR: 1.82; 95% CI: 1.57-2.12; *P* < 0.01; and OR: 1.93; 95% CI: 1.65-2.28; *P* < 0.01, respectively). These findings complement those from the Copenhagen General Population Study, which reported a 2- to 3-fold increased risk of PAD, AAA, and major adverse limb events at extremely elevated Lp(a) levels.^35^ However, those models did not adjust for ApoB, limiting their ability to distinguish Lp(a)-specific risk from risk mediated by ApoB-containing particles. This heightened risk for AAA parallels that of CAD, where an HR of 1.63 (95% CI: 1.56-1.70) has been reported in subgroups with high Lp(a) without existing ASCVD.^36^ These findings also align with the risk associated with major cardiovascular events (MACE), as genetic studies show an HR of 1.47 (95% CI: 1.43-1.52) for participants with median Lp(a) levels of 146.3 nmol/L compared with those with median levels of 13.6 nmol/L.^37^ These results suggest that the risk conferred by clinically high levels of Lp(a) on AAA mimics the effects seen across other vascular beds, including CAD and MACE, as supported by both observational and genetic studies.

Our observation that the relationship between Lp(a) and AAA was linear challenges the physiological relevance of current clinical trial thresholds used for outcome enrichment. This largely linear effect, with no evidence of a threshold effect across percentiles, has been previously demonstrated in studies exploring Lp(a) on incidence and risk of ASCVD. As shown by Patel et al,^36^ the adjusted HR of Lp(a) concentrations in the top quintile vs the middle quintile was 1.87 (95% CI: 1.68-2.08), with the same comparison done with LDL showing an adjusted HR of 2.20 (95% CI: 2.00-2.41). These findings align with more recent studies, in contrast to previous literature that emphasized the risk associated with Lp(a) is primarily restricted to those with very high concentrations.^38^

The concept of lowering Lp(a) regardless of the baseline is similar to the rationale for lowering LDL cholesterol. Lowering LDL-C significantly reduces cardiovascular events, as evidenced by multiple studies.^39^^,^^40^ For instance, early studies from the Cholesterol Treatment Trialists Collaboration found a 22% reduction in CV events with a 1-mmol/L (39 mg/dL) decrease in LDL-C, with benefits seen across all baseline levels, not just high levels of LDL-C.^40^ This evidence has been supported by multiple trials such as PROVE IT (Pravastatin or Atorvastatin Evaluation and Infection Therapy), TNT (Treating to New Targets), IMPROVE IT (IMProved Reduction of Outcomes: Vytorin Efficacy International Trial), and SPARCL (Stroke Prevention by Aggressive Reduction in Cholesterol), all showing that more intensive lowering of LDL-C significantly reduced cardiovascular events irrespective of thresholds.^41^, ^42^, ^43^, ^44^ These findings later translated into clinical guidelines, with the 2018 ACC/AHA and 2019 European Society of Cardiology guidelines, emphasizing that “lower is better” for LDL-C.^19^^,^^45^ Similarly, Burgess et al^46^ explored the risk reduction of Lp(a) using genetic techniques for CAD, indicating that the clinical benefit of lowering Lp(a) is proportional to the absolute reduction in its concentration. Based on our observation that the Lp(a) effects also appear linear for AAA, these findings suggest that substantial reductions in Lp(a) levels, irrespective of baseline levels of Lp(a), may translate into clinically meaningful improvements in AAA outcomes.

Last, we observe that the effects of Lp(a) on AAA are largely independent of ApoB. These findings suggest a potential role for Lp(a)-targeting medications in managing AAA in combination with ApoB-lowering medications like statins, which are recommended to reduce the concomitant risk of MACE among individuals with prevalent AAA.^47^ This finding is consistent with prior observations that Lp(a) is markedly more atherogenic than LDL.^48^ Mendelian randomization-derived OR analyses show that for CAD, a 50-nmol/L higher Lp(a)-apoB was 1.28 (95% CI: 1.24-1.33) compared with 1.04 (95% CI: 1.03-1.05) for the same increment in LDL-apoB. Further, the synergistic and independent roles of Lp(a) and ApoB have been highlighted in studies exploring cases of Lp(a) and familial hypercholesterolemia, showing that Lp(a) and familial hypercholesterolemia played a synergistic role in predicting the early onset and severity of CAD.^49^ Additionally, research leveraging data from both UKB and 23andMe has demonstrated that Lp(a) retains a causal effect on MACE even after accounting for LDL-C or apoB.^50^ Here, we demonstrate that this independent relationship of Lp(a) and ApoB extends to other vascular beds and remains true when considering the risk for AAA.

The therapeutic potential of Lp(a)-lowering for AAA, particularly in the context of other traditional risk factors, has remained uncertain.^51^ Ongoing trials such as OCEAN(a) and Lp(a)HORIZON have shown promising results regarding Lp(a) lowering, with a primary focus on cardiac events.^12^^,^^52^ Given the absence of approved pharmacological therapy for AAA, the linear association with no evident threshold effect, and the comparative risk profile to CAD at higher concentrations, Lp(a)-targeted therapy may be a viable for the prevention of AAA. This study highlights robust observational evidence supporting Lp(a)'s role in AAA pathobiology, reinforced by genetic analyses, suggesting a potential causal role that is independent of ApoB. These findings suggest that the inclusion of clinical endpoints related to AAA may be valuable to consider in ongoing Lp(a) lowering trials.

### Study limitations

This study should be analyzed within the context of its limitations. First, our observational study relied on data from the UKB, which may not be representative of the broader population caused by selection bias.^53^ Additionally, given the relatively low rate of AAA among UKB participants, we utilized prevalent, rather than incident, event analysis, which may have introduced some component of selection bias. However, because Lp(a) levels are not thought to vary substantially over time, this limitation may be less relevant.^54^

Additionally, although our findings support a largely linear relationship between Lp(a) and AAA risk, the clinical utility of treating individuals with lower baseline Lp(a) concentrations remains uncertain. Although relative risk reductions may be consistent across Lp(a) levels, the absolute benefit—and cost-effectiveness—of Lp(a)-lowering therapies will likely be much smaller in individuals with low baseline risk. This limits the justification for broad population-based treatment and underscores the importance of risk stratification when considering Lp(a)-targeted interventions for AAA prevention.

Second, several limitations of MR stem from its assumptions. Although we established that genetic variants selected as instrumental variables were strongly associated with the exposure of interest, MR additionally assumes that genetic variants influence the outcome solely through the exposure and not through other pathways, which may not always hold true in complex biological systems.^32^^,^^33^ By selecting genetic variants strongly associated with Lp(a) and ApoB, we satisfied the first assumption. Although efforts were made to minimize pleiotropy in genetic analyses, the presence of pleiotropic effects could still influence the results. Sensitivity analyses were conducted to address potential pleiotropy, presented in Supplemental Table 5, but residual confounding may still be present. Third, MR proxies the lifelong effects on variation within the physiologic range, in comparison to drug trials which consider more potent effects over a shorter duration which may not be represented through this study design.

The GWAS data for exposures and outcome, although described as multipopulation, were overwhelmingly of European ancestry, and while this minimizes confounding from population stratification, it may limit the generalizability of our findings to other ancestry groups. Further, a small proportion of participants overlapped, with UKB contributing to both the Lp(a)/ApoB GWAS and the AAA meta-analysis. Given the strong instruments used, any bias from this overlap is likely negligible. This is further supported by Supplemental Figure 2, which models bias at varying levels of sample overlap and shows minimal deviation even under complete overlap.^55^

## Conclusions

This study highlights, through both observational and genetic analyses, that Lp(a) confers a risk to AAA that is independent of other cardiovascular risk factors, including ApoB. Given the lack of medical therapy available in the management of AAA, this study underscores the potential role for Lp(a)-targeted therapy in future prevention and treatment of AAA.

Perspectives **COMPETENCY IN MEDICAL KNOWLEDGE:** Lp(a) is an independent risk factor for AAA development, distinct from other atherogenic lipoproteins such as ApoB. Understanding the role of Lp(a) in vascular pathology expands the mechanistic framework for AAA beyond traditional risk factors.

**COMPETENCY IN PATIENT CARE:** Measurement of Lp(a) may aid risk stratification in patients with or at risk for AAA, informing personalized monitoring and potential future therapeutic interventions.

**TRANSLATIONAL OUTLOOK:** Future research should prioritize prospective clinical trials to evaluate the efficacy of Lp(a)-lowering therapies in preventing AAA progression and rupture. Concurrently, implementation science studies are essential to identify and address barriers to widespread Lp(a) screening and the integration of Lp(a)-targeted therapies into clinical practice and AAA management guidelines. This dual approach will help accelerate the translation of genetic and observational findings into effective patient care strategies.

## Funding Support and Author Disclosures

This work was supported by the National Institutes of Health National Heart, Lung, and Blood Institute R01HL166991. Dr Levin was supported by the Doris Duke Foundation (Award 2023-0224) and U.S. Department of Veterans Affairs Biomedical Research and Development Award IK2-BX006551; receives research support to University of Pennsylvania from MyOme; and receives consulting fees from BridgeBio, outside the scope of this research. This publication does not represent the views of the Department of Veterans Affairs or the U.S. Government. Dr Damrauer receives research support to the University of Pennsylvania from RenalytixAI, and in kind support from Amgen and Novo Nordisk; and receives consulting fees from Tourmaline Bio, all outside the scope of this research.
