# Supplementary material for: Lipoprotein(a) Is Associated With Increased Risk of Abdominal Aortic Aneurysm
Source: JACC Basic Transl Sci. 2026 Jan 6;11(2):101457. doi: 10.1016/j.jacbts.2025.101457 (PMC12809141; doi:10.1016/j.jacbts.2025.101457)
Supplement: Supplemental Tables 1-6 and Supplemental Figures 1 and 2 [file mmc1.docx]

**Supplemental Appendix**

Table of Contents

1. Page 2: Supplemental Table 1. UK Biobank Data Fields
2. Page 3: Supplemental Table 2. Model summary per variable
3. Page 4: Supplemental Table 3. Model summary per variable (excluding high and low values)
4. Page 5: Supplemental Table 4. Total Harmonized Data For Univariable MR (LPA and APOB)
5. Page 6: Supplemental Table 5. Results of Mendelian randomization and Sensitivity Analyses for Lp(a) and ApoB
6. Page 7: Supplemental Table 6. Total Harmonized Data For Multivariable MR
7. Page 8: Supplemental Figure 1. Observational Analyses of Lp(a) Concentration and Risk of Abdominal Aortic Aneurysm Excluding Extreme Values
8. Page 9: Supplemental Figure 2. Simulation of bias and Type 1 error at varying levels of sample overlap for Lp(a) and AAA MR analysis

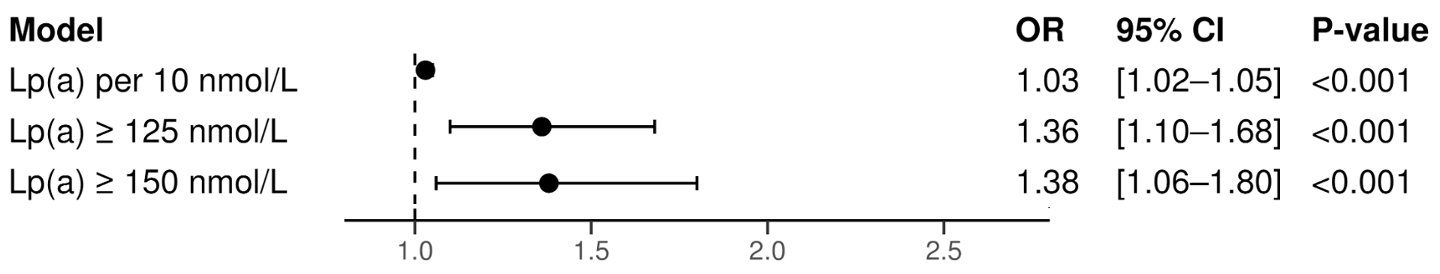


**Supplemental Figure 1. Observational Analyses of Lp(a) Concentration and Risk of Abdominal Aortic Aneurysm Excluding Extreme Values.** This figure presents three observational models assessing the association between Lp(a) and AAA risk, after excluding individuals with extreme Lp(a) values (>189 nmol/L and <3.9 nmol/L). Models include: (1) per 10 nmol/L increase in Lp(a), (2) Lp(a) ≥125 nmol/L vs. <125 nmol/L, and (3) Lp(a) ≥150 nmol/L vs. <150 nmol/L. Results are shown as ORs with 95% CIs. All models were adjusted for age, sex, race/ethnicity, ApoB, ApoA1, diabetes, smoking, hypertension, LDL cholesterol, and HDL cholesterol. Abbreviations: AAA = abdominal aortic aneurysm; ApoA1 = apolipoprotein A1; ApoB = apolipoprotein B; CI = confidence interval; HDL = high-density lipoprotein; LDL = low-density lipoprotein; Lp(a) = lipoprotein(a); OR = odds ratio.

**
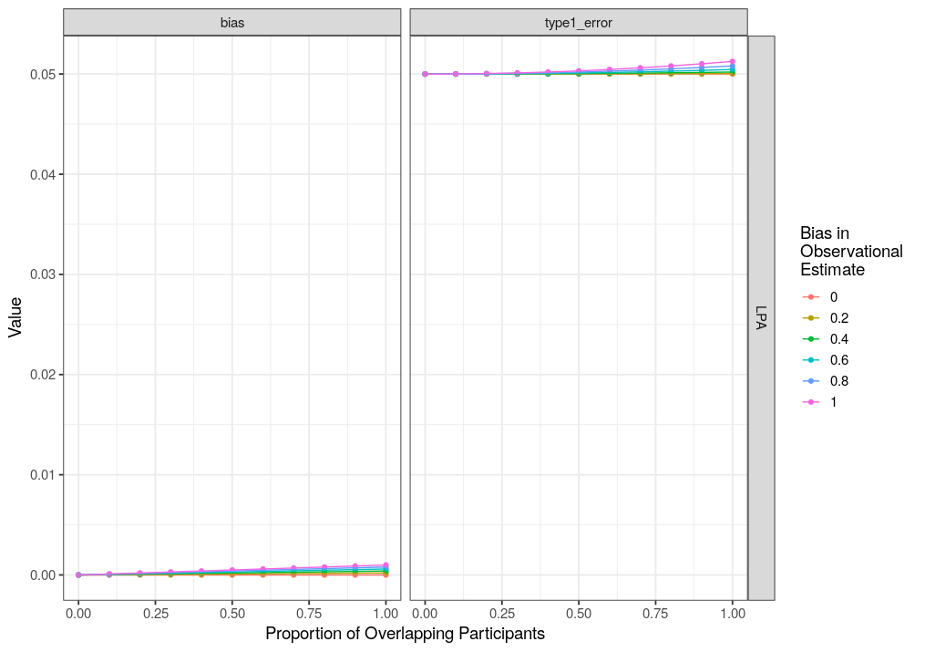
**

**Supplemental Figure 2. Simulation of bias and Type 1 error at varying levels of sample overlap for Lp(a) and AAA MR analysis.** This figure highlights a simulation-based assessment of bias and type I error under varying proportions of overlapping participants between exposure and outcome GWAS using the framework described by Burgess et al. Abbreviations: LPA = lipoprotein(a).
